# Age- and cause-specific contributions to increase in life expectancy at birth in Korea, 2000–2019: a descriptive study

**DOI:** 10.1186/s12889-024-17974-4

**Published:** 2024-02-10

**Authors:** Ikhan Kim, Hyeona Bae

**Affiliations:** Department of Medical Humanities and Social Medicine, Kosin University College of Medicine, 262 Gamcheon-ro, Seo-gu, Busan, 49267 Korea

**Keywords:** Causes of death, Chronic disease, Communicable diseases, Life expectancy, Life tables, Republic of korea

## Abstract

**Background:**

Korea’s life expectancy at birth has consistently increased in the 21st century. This study compared the age and cause-specific contribution to the increase in life expectancy at birth in Korea before and after 2010.

**Methods:**

The population and death numbers by year, sex, 5-year age group, and cause of death from 2000 to 2019 were acquired. Life expectancy at birth was calculated using an abridged life table by sex and year. The annual age-standardized and age-specific mortality by cause of death was also estimated. Lastly, the age and cause-specific contribution to the increase in life expectancy at birth in the two periods were compared using a stepwise replacement algorithm.

**Results:**

Life expectancy at birth in Korea increased consistently from 2010 to 2019, though slightly slower than from 2000 to 2009. The cause-specific mortality and life expectancy decomposition analysis showed a significant decrease in mortality in chronic diseases, such as neoplasms and diseases of the circulatory system, in the middle and old-aged groups. External causes, such as transport injuries and suicide, mortality in younger age groups also increased life expectancy. However, mortality from diseases of the respiratory system increased in the very old age group during 2010–2019.

**Conclusions:**

Life expectancy at birth in Korea continued to increase mainly due to decreased mortality from chronic diseases and external causes during the study period. However, the aging of the population structure increased vulnerability to respiratory diseases. The factors behind the higher death rate from respiratory disease should be studied in the future.

**Supplementary Information:**

The online version contains supplementary material available at 10.1186/s12889-024-17974-4.

## Background

South Korea (hereafter, Korea) has experienced consistent improvement in life expectancy at birth (LE) in recent years. According to a previous study, the LE of Korea increased by 16.4 years in men and 16.3 years in women from 1970 to 2005, which was far better than the average of Organisation for Economic Co-operation and Development (OECD) countries [1]. Reduction of infant mortality and cardiovascular diseases (CVD), liver disease, stomach cancer, tuberculosis, pneumonia, and external-cause mortalities contributed to the increase in LE [1]. Other studies revealed that from 1998 to 2017, LE in Korea steadily increased, and the decrease in avoidable causes of death, such as CVD, neoplasms, and transport injuries, contributed the most in the period [2, 3]. It was projected that if this trend continues, by 2030, the LE in Korea, particularly among women, will be the highest globally [4]. Nonetheless, it is known that Korea has recently seen a rise in the mortality rate from infectious diseases [5, 6].

This increase in LE in Korea contrasts with the stagnation in LE increase seen in several other countries since the 2010s [7]. For example, in the United Kingdom, LE increased for men and women by 1.7 and 1.4 years, respectively, from 2006 to 2011 and by 0.4 and 0.0 years from 2011 to 2016 [7]. This trend has been attributed to increased mortality due to dementia, respiratory diseases, and cardiovascular and circulatory diseases (CVD) in the aging population [8]. In the United States, LE decreased from 78.9 years in 2014 to 78.7 years in 2018 [9]. This decline is suspected to be due to a slowdown in improving CVD mortality and rising mortality from drug overdoses, suicide, homicide, and dementia [10].

Omran argued that since chronic diseases have replaced infectious diseases as the major CaD, the rise in LE in many countries results from substituting infant mortality for death in old age [11]. Olshansky et al. added a fifth stage, which relates to the recurrence of infectious diseases, and the fourth stage, which asserts that death from degenerative diseases continues to be delayed in the older age group [12, 13]. While epidemiologic transition processes vary across countries, examining changes in LE in a specific country and contrasting similarities and differences between countries might help us better understand changes in LE [14]. Understanding the pattern would also allow us to make more informed projections on how the LE of Korea may change.

This study aimed to calculate the recent changes in LE and cause-specific mortality and quantify the age and cause-specific contribution to the LE increase. This study compared the first decade’s (2000–2009) changes in LE and cause-specific mortality with those of the second decade (2010–2019). In addition, this study estimated and compared age- and cause-specific contributions to LE changes in each decade.

## Methods

### Data

We acquired the population and death by year (2000–2019), sex, 5-year age group (0, 1–4…95–99, 100+), and cause of death (CaD) from Statistics Korea. Statistics Korea generates various statistics, including annual mid-year resident population and death statistics. Death statistics provide various information such as sex and age, date of death, district of residence, and CaD. The cause of death was determined using the Korean Standard Classification of Diseases and Causes of Death, Seventh Revision, based on the International Classification of Diseases, Tenth Revision [15]. We adjusted the classified individual CaD into 15 major categories, referring to the World Health Organization classification [16]. Since life expectancy started to decline or stagnate in many other countries in 2010, we separated the entire study period into two periods: 2010–2019 and 2000–2009 [7].

#### Outcomes

LE and age-standardized mortality were outcomes of this study. LE is a summary measure calculated using a series of formulas to determine how many years infants born in the current year will live if they experience the mortality rate by age group in the current year [17]. Age-standardized mortality refers to the mortality assumed when the age structure is the same as the standard population to adjust for the differences in the age structure in each year.

### Statistical analysis

We conducted all analyses stratified by sex. Following the well-established approach, we constructed the abridged life tables and estimated LE by year and sex [17]. The Monte Carlo simulation design was used to obtain the 95% uncertainty intervals (95% UI). The number of deaths by year, sex, CaD, and age group were assumed to have a Poisson distribution. Two thousand values were randomly drawn from the corresponding Poisson distribution. The median value of LE estimated using 2,000 death numbers was set as the point estimate, and the 2.5 percentile and 97.5 percentile values were designated as the lower and upper limits for the 95% UI. The open-age interval was set as 100 years and older. Then, we calculated and compared LE’s absolute and relative increase during 2000–2009 and 2010–2019.

A previous study has reported that it is preferable to present changes in mortality rates along with changes in LE [18]. We estimated the directly age-standardized cause-specific mortalities by year and sex. The population of Korea as a whole in 2010 was used as the standard population to estimate the age-standardized cause-specific mortalities. Then, using the following equation, we compared the absolute differences in cause-specific mortalities between 2000 and 2009 and 2010–2019.

$$ \textit{diff}_{\text{2000,2009}}={mor}_{2000}-{mor}_{2009}$$

$$ \textit{diff}_{\text{2010,2019}}={mor}_{2010}-{mor}_{2019}$$

where $$ \textit{diff}$$ refers to the absolute differences in age-standardized cause-specific mortalities between the first (2000 and 2010) and last year (2009 and 2019) of each period. $$ mor$$ refers to the age-standardized cause-specific mortality of a specific year (2000, 2009, 2010, and 2019). Therefore, positive differences in cause-specific mortality show a decrease in cause-specific mortality and vice versa. We also estimated changes in mortality rates by age group and CaD from 2000 to 2019. We adjusted age groups into five new age groups (0–14, 15–39, 40–64, 65–79, 80+) and six death causes (neoplasms, diseases of the circulatory system, diseases of the respiratory system, diseases of the digestive system, external causes, and others) for clarity of results.

We used the standard stepwise replacement algorithm to decompose LE changes by age group and death cause in two interdecadal periods [19]. First, we replaced the age group (0–14, 15–39, 40–64, 65–79, 80+) and cause-specific (neoplasms, diseases of the circulatory system, diseases of the respiratory system, diseases of the digestive system, external causes, and others) mortality of 2000 and 2010 with that of 2009 and 2019, respectively. Then, we calculated the gap between the LE values before and after replacement, which referred to the age group and cause-specific contribution to the LE increases during the period. Lastly, we differentiated the age group and cause-specific contribution in the first period (2000–2009) from the second period (2010–2019). The statistical software R version 4.2.1 (https://www.r-project.org/) was used for analysis.

## Results

**Fig. 1:**
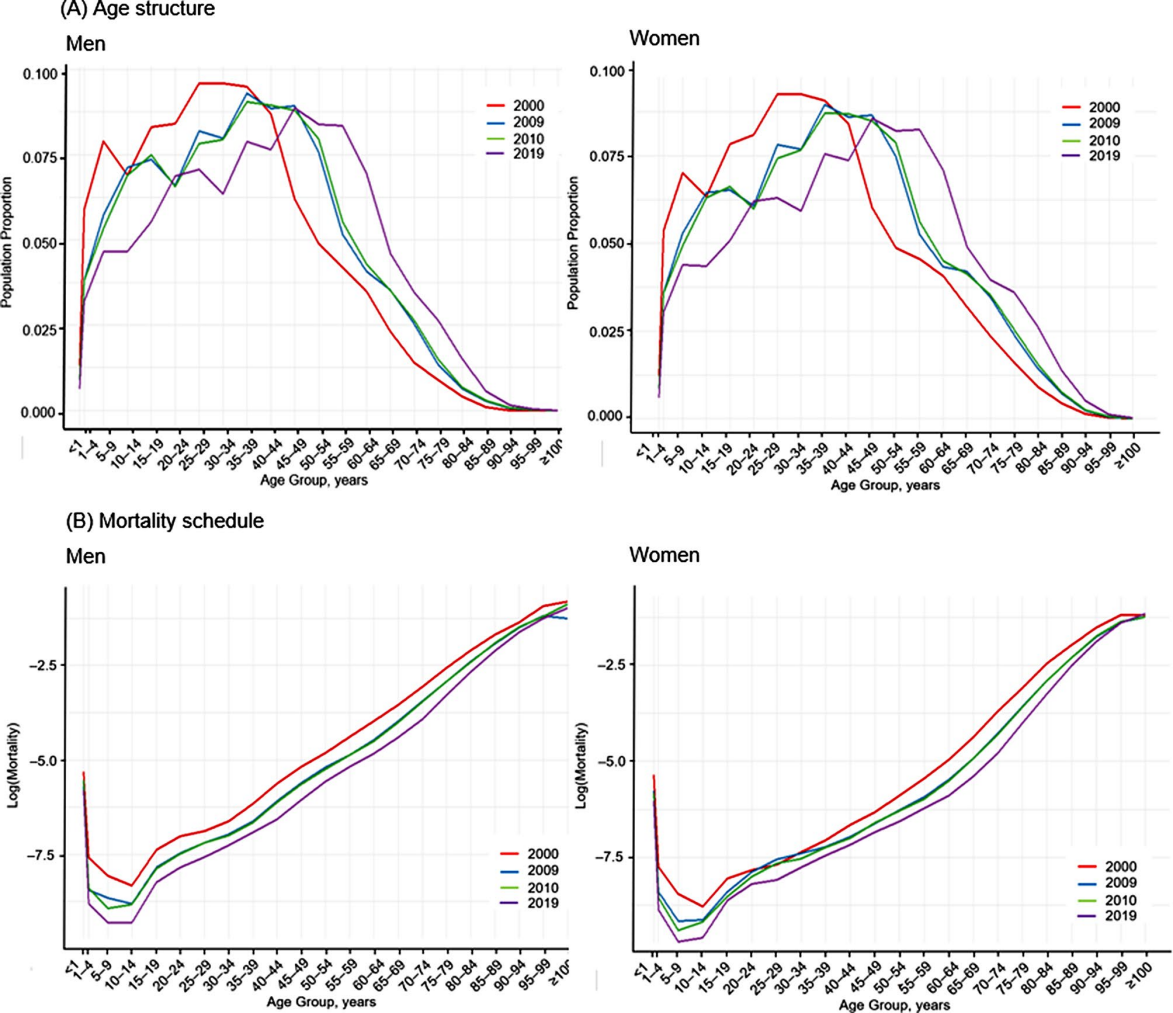
Age structure and mortality schedule in korea in 2000, 2009, 2010, and 2019 by sex: Findings from korea statistical information service (KOSIS)

Fig. 1 shows the pattern of age structure and log-transformed mortality schedule changes by sex across the study period. Most of the population belonged to the young and middle-aged groups, and as time progressed, both sex’s age structures grew. The log-transformed age-specific mortality formed a V shape and declined across all age groups during the study period.

Table 1 and Supplementary Fig. 1 show the sex-specific changes in LE over the entire study. The men’s LE was 72.3 (95% UI: 72.2–72.4) years in 2000, while the women’s LE was 79.6 (95% UI: 79.6–79.7). By 2009, the men’s LE had increased by 6.3% (4.5 years), and the women’s LE by 5.1% (4.1 years). In 2010, the men’s LE was 77.0 (95% UI: 76.9–77.0) years, and the women’s LE was 83.9 (95% UI: 83.8–84.0) years. The men’s LE had grown by 4.5% (3.5 years) by 2019, and the women’s LE had expanded by 3.2% (2.7 years).

**Table 1:** The absolute and relative increase in life expectancy of Korea during the 2000–2009 and 2010–2019 periods by sex: Findings from the korea statistical information service (KOSIS)

	2000–2009				2010–2019			
	LE in 2000, yrs (A)	LE in 2009, yrs (B)	Absolute increase, yrs (B)-(A)	Relative increase, % (B)-(A) / (A)	LE in 2010, yrs (A)	LE in 2019, yrs (B)	Absolute increase, yrs (B)-(A)	Relative increase, % (B)-(A) / (A)
Men	72.3 (72.2, 72.4)	76.9 (76.8, 76.9)	4.5 (4.5, 4.6)	6.3 (6.2, 6.4)	77.0 (76.9, 77.0)	80.5 (80.4, 80.5)	3.5 (3.4, 3.6)	4.5 (4.4, 4.6)
Women	79.6 (79.6, 79.7)	83.7 (83.6, 83.8)	4.1 (4.0, 4.2)	5.1 (5.0, 5.2)	83.9 (83.8, 84.0)	86.6 (86.6, 86.7)	2.7 (2.6, 2.8)	3.2 (3.1, 3.4)

*Notes* LE = Life expectancy

The lower and upper limits of the 95% uncertainty interval are in parentheses

**Fig. 2:**
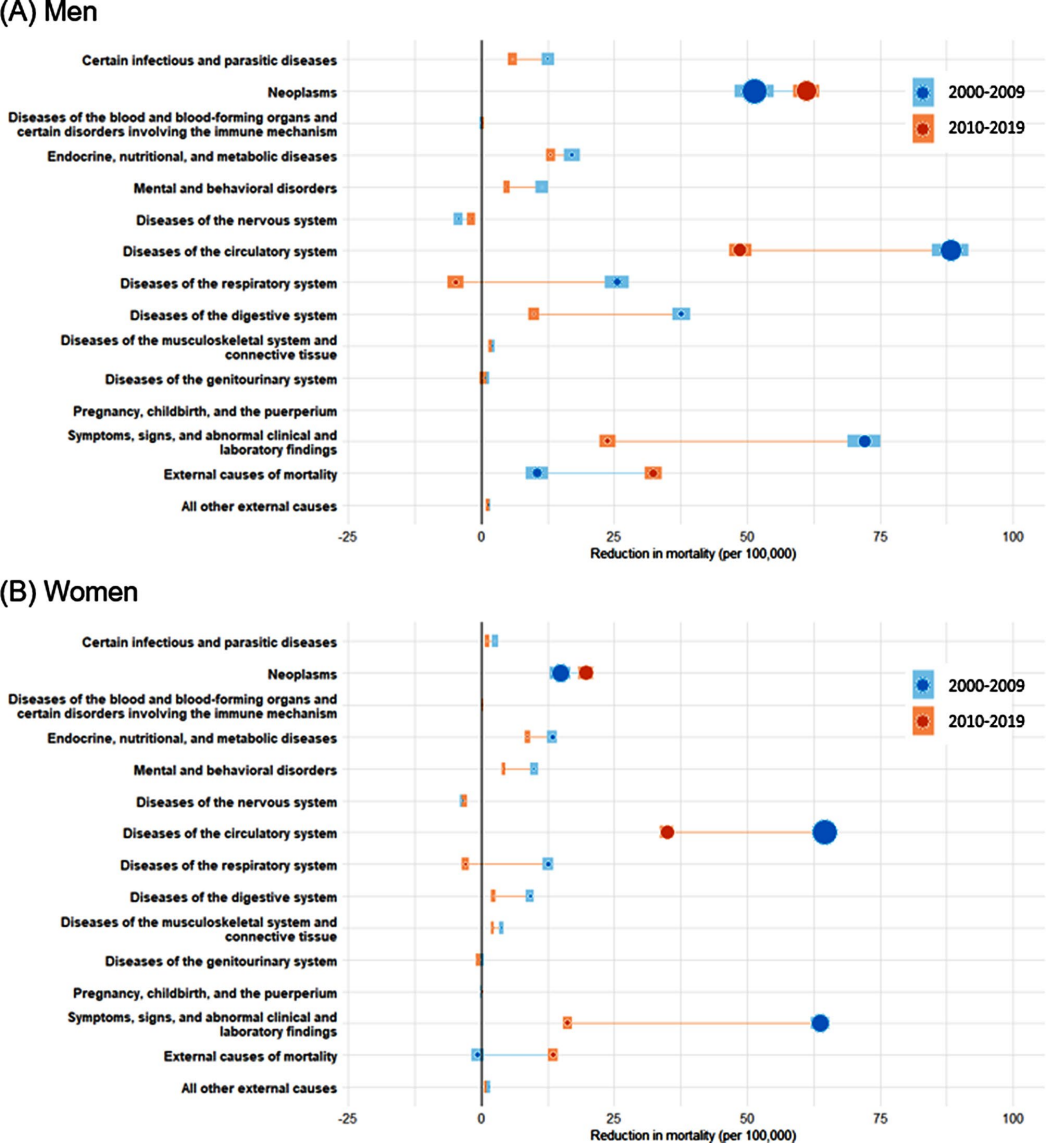
Comparison of absolute changes in age-standardized cause-specific mortality between 2000–2009 and 2010–2019 by sex: Findings from korea statistical information service (KOSIS). (**a**) Standard population was the total population of Korea in 2010. (**b**) Positive values indicate decreases in mortality, and negative values indicate increases in mortality during a specific period. (**c**) The size of point estimates varied depending on the mortality rate in 2000 and 2010. (**d**) The area of the shading indicates the 95% uncertainty interval

The absolute decrease in cause-specific mortality for (A) men and (B) women between the two periods is described in Fig. 2. Neoplasms and diseases of the circulatory system mortality significantly declined in both sexes in 2000–2009 and 2010–2019. In addition, digestive system diseases and external causes of mortalities decreased in men from 2000 to 2019. From 2010 to 2019, the magnitudes of decrease were smaller in almost all diseases except for neoplasms and external causes of mortality. Women shoed similar tendencies to those of men. Notably, the 2010s have seen a decline in mortality due to external causes, which had risen in the 2000s. Mortality from diseases of the respiratory system and nervous system in both men and women rose in the 2010s. Supplementary Fig. 2 depicts the trends in age-standardized cause-specific mortality by sex during the study period.

**Fig. 3:**
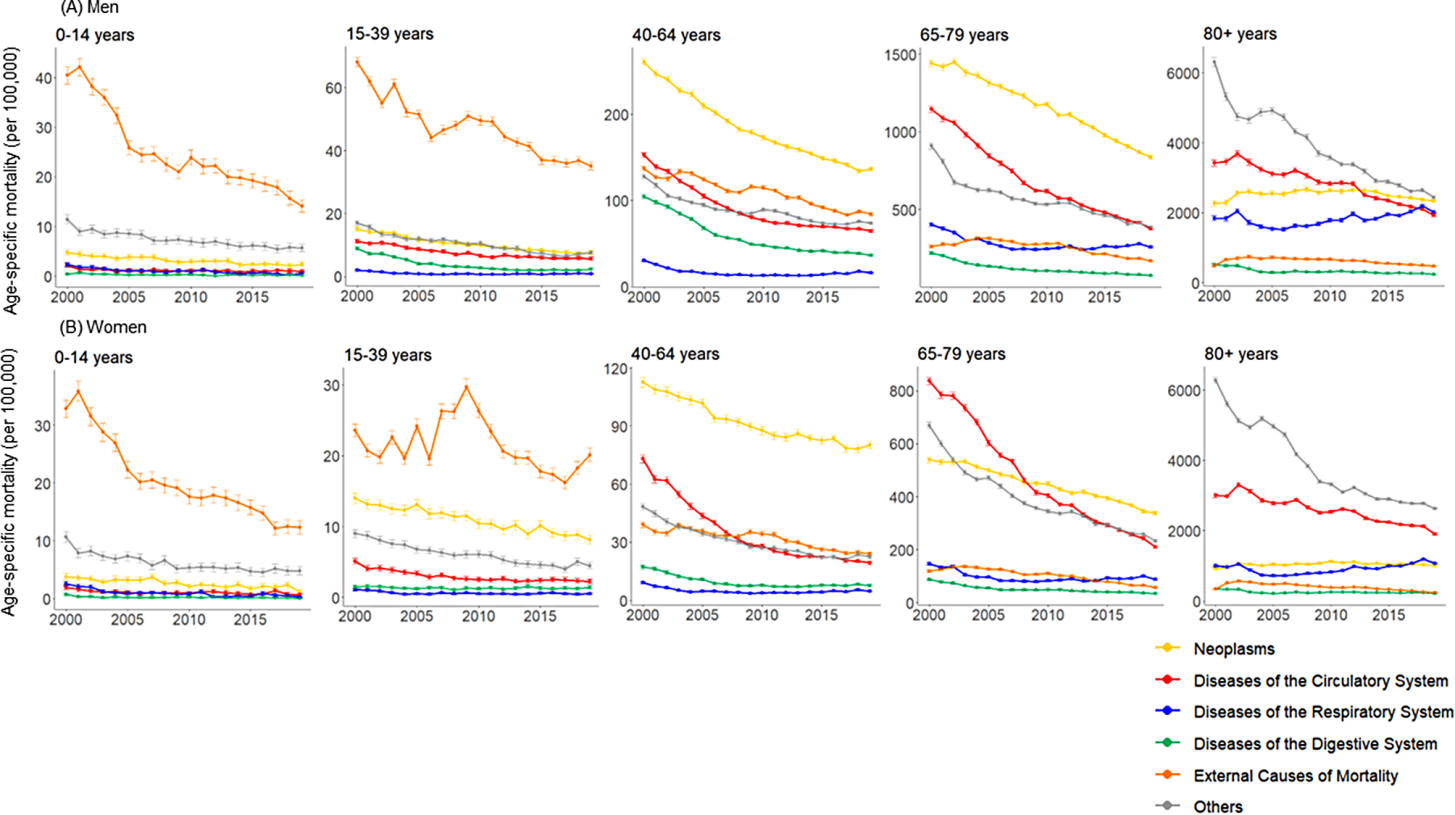
Annual trend in cause-specific mortalities by sex and age group, 2000–2019: Findings from korea statistical information service (KOSIS). The figure’s point estimate is indicated by the point, and the 95% uncertainty interval is shown by the bar that surrounds the point

In Fig. 3, the change in the mortality rate from CaD by sex and age group in Korea between 2000 and 2019 is graphically depicted. In the 0–14 and 15–39 age groups, there was a dramatic decrease in men’s external causes of mortality, particularly in the 2000–2009. The mortality rate from circulatory system diseases and neoplasms in the 40–64 and 65–79 year old age groups decreased significantly. In the 65 and older age groups, there was also a discernible decline in mortality from other causes of death. Similar to men, women saw a significant decrease in external causes of death in the 0–14 age group but not in the 15–39 age group. Among women, there was a notable decrease in mortality from circulatory system diseases in the 40–64 and 65–79 age groups. In the age group of 65 years or older, mortality from other causes of death decreased significantly, similar to the situation with men.

**Fig. 4:**
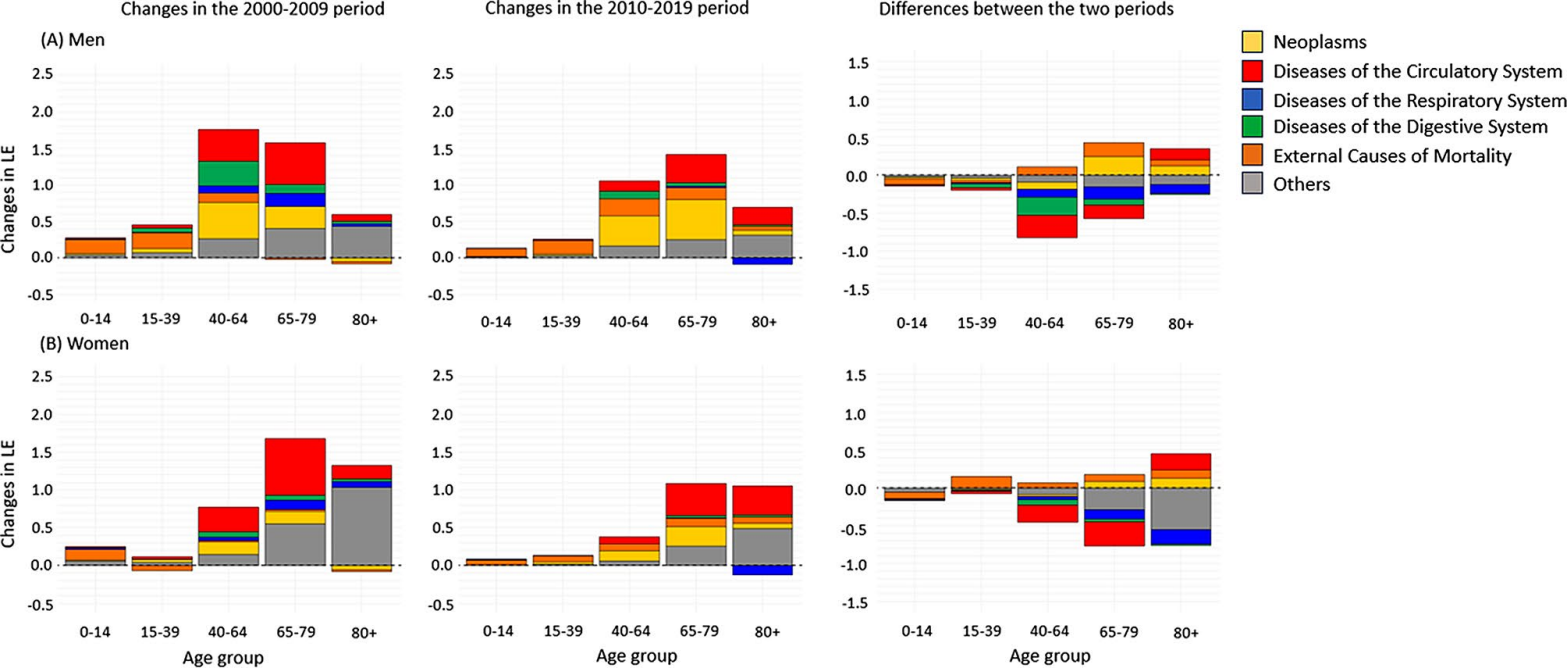
Comparison of age- and cause-specific contribution to life expectancy increase in 2000–2009 and 2010–2019 period in Korea by sex: Findings from korea statistical information service (KOSIS). LE = life expectancy. (**a**) Positive values indicate increases in LE, and negative values indicate decreases in LE during a specific period. (**b**) The change from 2000 to 2009 was subtracted from the change from 2010 to 2019 to estimate the difference between the two periods

Fig. 4 shows the results of the decomposition analysis by sex. By age group, the increase in LE was contributed mainly by reducing mortality among those in their 40s or older. According to CaD, the increase in LE in men was contributed, in that order, by others (1.2 years), circulatory system diseases (1.1 years), neoplasms (0.8 years) between 2000 and 2009, and diseases of the circulatory system (0.9 years), others (0.8 years), and neoplasms (0.5 years) between 2010 and 2019. In women, the most significant contributions between 2000 and 2009 came from others (1.8 years), circulatory system diseases (1.3 years), and neoplasms (0.3 years). From 2010 to 2019, the most significant contributions came from circulatory system diseases (0.9 years), others (0.8 years), and neoplasms (0.5 years), in that order. External causes contributed the most to young age groups for both men and women. Chronic diseases such as neoplasms and diseases of the circulatory system contributed to the consistent increase in LE in 2010–2019 in the age group over 40 in both sexes, though relatively more in the very old age group in women. However, mortality of respiratory system diseases contributed to a decrease in LE in the age group 80+.

## Discussion

This study found that LE in Korea consistently increased between 2000 and 2019. The improvement of LE in both decades was driven by declined mortality in neoplasms and CVD among old and very old age groups in both sexes. External causes in younger age groups also contributed to the increase in LE. However, respiratory disease mortality in those aged over 80 increased in the recent decade.

In contrast to several other countries, LE increased steadily in Korea over the study period. The result is consistent with other studies [1–3]. According to a recently released research that examined changes in population-level health outcomes in Korea, the country’s age-standardized mortality rate fell by 57.9% for both men and women between 1990 and 2019 [20]. Specifically, the authors reported that the decline in mortality for non-communicable diseases was the vital cause of Korea’s declining mortality rate [20]. A recent study, however, revealed that several European nations, as well as some other countries, including the United States, Australia, and Canada, have seen a decrease in the rate of LE increase since 2011 [7, 10]. The rise in LE in the US has stagnated or even declined since 2010, according to a study that examined trends in LE by sex, race and ethnicity, socioeconomic status, and geography from 1999 to 2018 [10]. Men and those in the 15–44 age group significantly contributed to this change [10].. On the other hand, some East Asian nations are seeing a comparatively constant rise in LE [21, 22]. In Japan, the increase in LE, which decreased significantly from 2007 to 2011, showed a significant increase from 2012 to 2016 [23]. However, a study comparing life expectancy at age 60 between Japan and Korea reported that the difference in life expectancy at age 60 between the two countries decreased by 2.47 years from 1997 to 2017 [2]. The two countries’ leading causes of the difference in life expectancy at age 60 were neoplasms and CVD [2].

An important driver of the increase in LE in Korea was the declining mortality in chronic diseases such as CVD and neoplasm in the older groups. Most countries in Norway and some East Asian countries have similarly seen this tendency, where LE has continued to increase [21, 22, 24]. Conversely, the rate of improvement for neoplasm mortality and CVD has decreased since 2003, which has also halted the rapid LE increase in Australia [25]. The substantial decrease in CVD mortality that began in the 1960 and 1970 s induced LE to increase in many countries [26]. However, significant decreases in CVD mortality in Europe and the United States have reached a standstill [27]. For instance, the annual decline in CVD mortality in the United States has been approximately 1% since 2010, compared to 3.5% in the prior decade [27]. Even before 2010, Korea had the highest rate of decline in men’s and women’s CVD mortality, at 6.3% and 8.4%, respectively [27]. Additionally, only Korea and Singapore had decrease rates in 2017 that were higher than 2% [27]. The annual mortality decline rate for neoplasms worldwide was roughly 1% from 2000 to 2010 [28]. In Korea, it fell at a pace of 2.7–2.9% over the same period [28]. Risk factors, including smoking prevalence, hypertension, diet, and body weight, and advances in evidence-based medicine, emergency medicine, and screening significantly lowered mortality from chronic diseases like CVD and neoplasms [29, 30]. Given that most subcategory mortalities declined, various factors may have contributed to the decrease in mortality in Korea for CVD and neoplasms (Results available on request). For example, once tobacco control legislation was implemented after the 2000s, men’s smoking rates in Korea drastically fell [31]. In addition, the average blood pressure, awareness, and treatment of high blood pressure have greatly improved [31]. Furthermore, the national cancer screening program for neoplasms may improve access to medical care in Korea [32].

The increase in LE of the younger age group in Korea was attributed mainly to external causes such as transport injuries, suicide, and interpersonal violence. Rogers & Hackenberg classified deaths caused by accidents, suicide, homicide, alcoholism, and liver cirrhosis as a social pathology, which varies widely among countries [33]. According to a recent study, one of the main CaD for people between the ages of 15 and 44 is injuries from falls and transport accidents [34]. Until the middle of the 1990s, Korea’s transport injury mortality rate rose dramatically along with the country’s economic growth [35]. A special national commission for traffic safety was established in 1999, and several new government regulations, including mandatory seat belt use and the maximum speed limit for cars, have been implemented [35]. As a result, the mortality rate associated with transport injuries was reduced by roughly 6.1% annually before 2015 and 12.9% after 2015 [36].

We mainly interpreted the results as a decline in suicide mortality since the mortality rate due to interpersonal violence in Korea is relatively low compared to the mortality rate due to suicide (25 times difference) [36]. From 1986 to 2005, the suicide mortality rate in Korea climbed by 124% for women and 98% for men, making it one of the OECD countries with the highest suicide rate [37]. In particular, older age groups had much higher suicide rates than younger age groups, which was thought to be related to economic hardship [38]. The suicide rate and unemployment were associated with younger age groups [39]. Korea’s 2nd Five-Year Suicide Prevention Plan (2008–2012), which started in 2008, expanded the stakeholder range and intervention range—for example, installing subway screen doors or regulating the use of paraquats [40]. A basic old-age pension policy for older people was implemented in 2008, and the pension amount continuously increased [41]. According to one study, suicide mortality rose by 5.08% annually between 2005 and 2010 but fell by 4.46% between 2010 and 2017 [36]. 2017 saw the lowest suicide rate in recent decades despite the reality that it still has one of the worst rates among OECD countries [42].

The persistent increase in LE in Korea could result from a decline in regional inequalities in LE. The rise in LE between 2000 and 2009 and 2010–2019 would have been 0.5 and 0.4 years less if the mortality rate by age group in Korea’s rural and urban areas had declined at the same rate as in metropolitan areas, according to a previous study [43]. LE in Norway continued to increase between 2000 and 2019, coinciding with a decrease in the LE gap between counties [24]. The reduction in CVD, neoplasms, and respiratory infections contributed significantly [24]. On the other hand, since 2011–12, LE growth has stalled in Wales, accompanied by an increase in aerial LE inequality after a fall in LE in areas with high levels of deprivation [44]. Respiratory, digestive, and circulatory disorders, malignancies, dementia, and substance abuse were the CaD that significantly increased LE inequality [44].

The study’s findings indicate that the decline in LE was caused by increased respiratory disease mortality in the 80 and older age group between 2010 and 2019. Previous studies found that although total mortality in Korea decreased between 1983 and 2015, mortality from infectious diseases increased again since 2015, and the proportion of infectious deaths in total deaths also increased [5, 6]. The increase in respiratory disease mortality is thought to be caused by an increase in the old population over 65 who live with chronic conditions, which is believed to lead to a rise in the number of people who die from infectious diseases in a state of weakened immunity [5]. Additionally, it’s considered that a rise in nosocomial infections and antibiotic resistance due to the number of individuals who spend their final years in communal environments like nursing institutions has had an impact [5].

### Limitations

This study has the following limitations. First, some of the CaD designations may be inaccurate. The CaD is determined following medical confirmation in almost all cases of death data in Korea. Nevertheless, the issue of ill-defined CaD has been raised, and the subject of categorizing causes of death has been addressed in several ways [45, 46]. In a previous Korean study, 24.6% of all deaths in 2010 (21.0% men, 29.2% women) were classified as garbage codes [45]. An earlier European study revealed that as of 2017, 22–31% of all deaths in those countries were accounted for by garbage codes [47]. This study also found that, despite a sharp decline since the early 21st century in the mortality rate from causes of death categorized as “symptoms, signs, and abnormal clinical and laboratory findings,” over 20 per 100,000 persons died from these causes in 2019. As a result, others of CaD contributed significantly to the increase in LE. Second, the study’s population and death data sets were unlinked. Numerator-denominator bias is known to emerge when unlinked death and population data are used. The use of linked or unlinked data affected the result of education-specific life expectancy trends [48, 49]. However, because this study examined national-level LE trends, it may induce lesser numerator-denominator bias than if data were used at the province or district levels. Furthermore, previous Korean studies that used linked data also showed consistently increased LE trends, as demonstrated by this study [50, 51].

## Conclusions

This study found that even in the 2010s, LE in Korea rose steadily. The decrease in mortality from chronic diseases such as neoplasms and CVD in the older age group and external mortality played a significant role in the consistent LE increase. These findings suggest that the recent rise in LE in Korea may have been greatly affected by societal capability and policy initiatives. However, respiratory disease mortality among those over 80 contributed to a decrease in LE in the 2010s. Future studies on the factors contributing to the rise in respiratory disease-related deaths, as well as political efforts to address these issues, are needed.

### Electronic supplementary material

Below is the link to the electronic supplementary material.

**Supplementary Material 1: Supplementary Fig. 1.** Life expectancy change by sex in Korea, 2000-2019: Findings from Korea Statistical Information Service (KOSIS). **Supplementary Fig. 2.** Trends in age-standardized cause-specific mortality by sex during the study period: Findings from the Korean Statistical Information Service (KOSIS)

## Data Availability

The datasets analyzed during the current study are publicly available through the National Statistical Portal of the Korean Statistical Information Service (https://kosis.kr/index/index.do).

## Abbreviations

CaD: cause of death
CVD: cardiovascular diseases
LE: life expectancy at birth
OECD: Organisation for Economic Co-operation and Development
95% UI: 95% uncertainty interval
