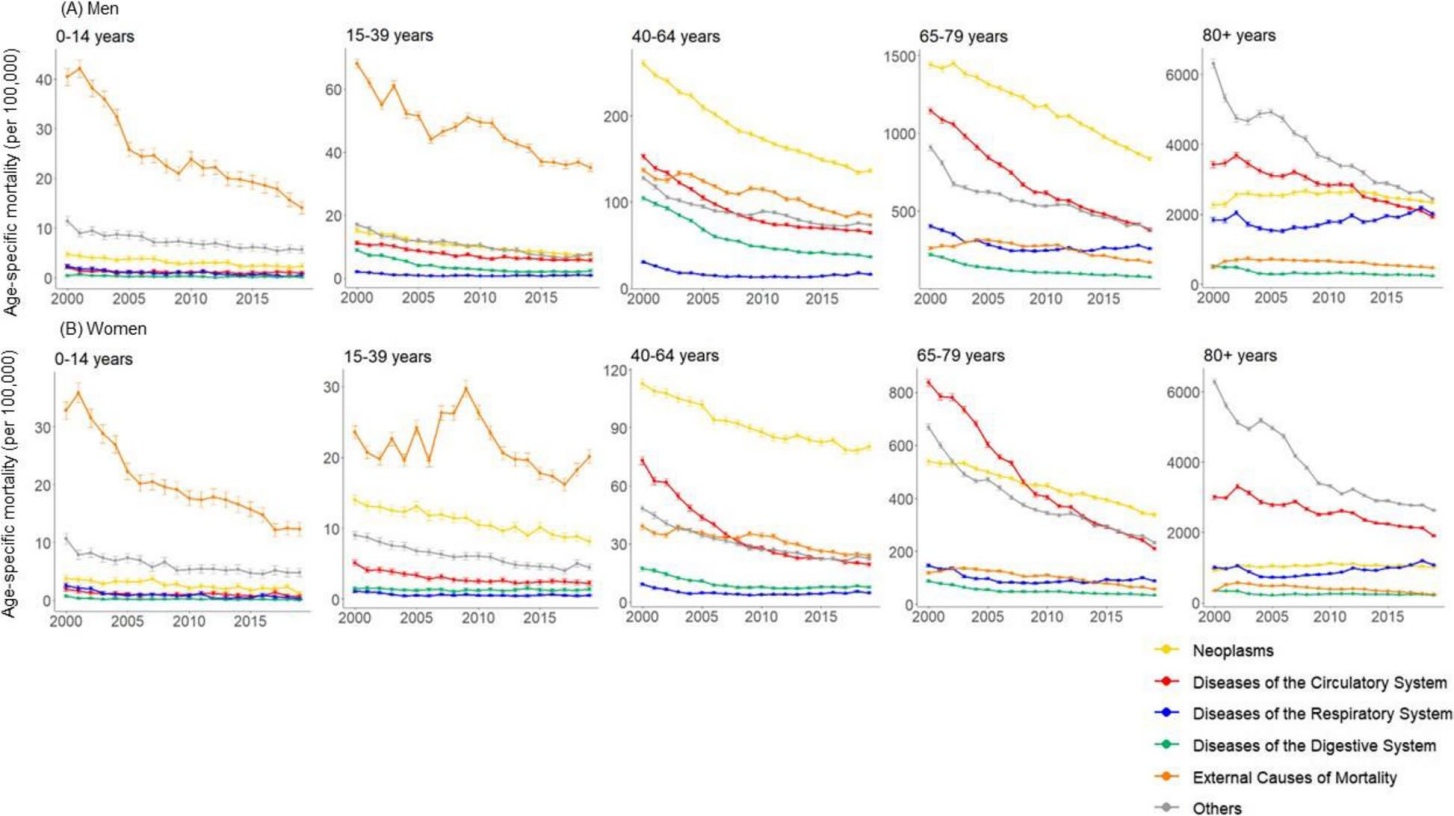# Correction: Age- and cause-specific contributions to increase in life expectancy at birth in Korea, 2000–2019: a descriptive study

**DOI:** 10.1186/s12889-024-19019-2

**Published:** 2024-06-03

**Authors:** Ikhan Kim, Hyeona Bae

**Affiliations:** Department of Medical Humanities and Social Medicine, Kosin University College of Medicine, 262 Gamcheon-Ro, Seo-Gu, Busan, 49267 Korea

**Correction** **:** **BMC Public Health 24, 431 (2024)**

**https://doi.org/10.1186/s12889-024-17974-4**

In the original publication of this article [1] there was an error in Figure 3. The mortality rate by gender, age group, and cause of death presented in figure 3 was presented as number of deaths per 100,000 people. However, the death rate was overestimated as a result of a computation error. and as a result, adding up the number of deaths by cause of death exceeds 100,000 in the oldest age group.

The incorrect and correct figure 3 are shown in this correction article. The original article has been updated.

**Incorrect figure 3**


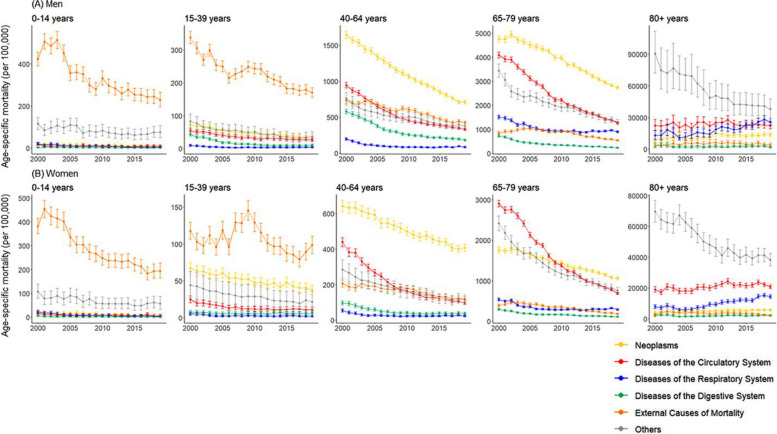


**Correct figure 3**