# Supplementary material for: Age- and cause-specific contributions to increase in life expectancy at birth in Korea, 2000–2019: a descriptive study
Source: BMC Public Health. 2024 Feb 10;24:431. doi: 10.1186/s12889-024-17974-4 (PMC10859017; doi:10.1186/s12889-024-17974-4)
Supplement: Supplementary file 1 — Supplementary Material 1: Supplementary Fig. 1. Life expectancy change by sex in Korea, 2000-2019: Findings from Korea Statistical Information Service (KOSIS). Supplementary Fig. 2. Trends in age-standardized cause-specific mortality by sex during the study period: Findings from the Korean Statistical Information Service (KOSIS) [file 12889_2024_17974_MOESM1_ESM.docx]

**Supplementary Materials**

**Supplementary Fig. 1.** Life expectancy change by sex in Korea, 2000-2019: Findings from Korea Statistical Information Service (KOSIS).

Supplementary Fig. 2. Trends in age-standardized cause-specific mortality by sex during the study period: Findings from the Korean Statistical Information Service (KOSIS).


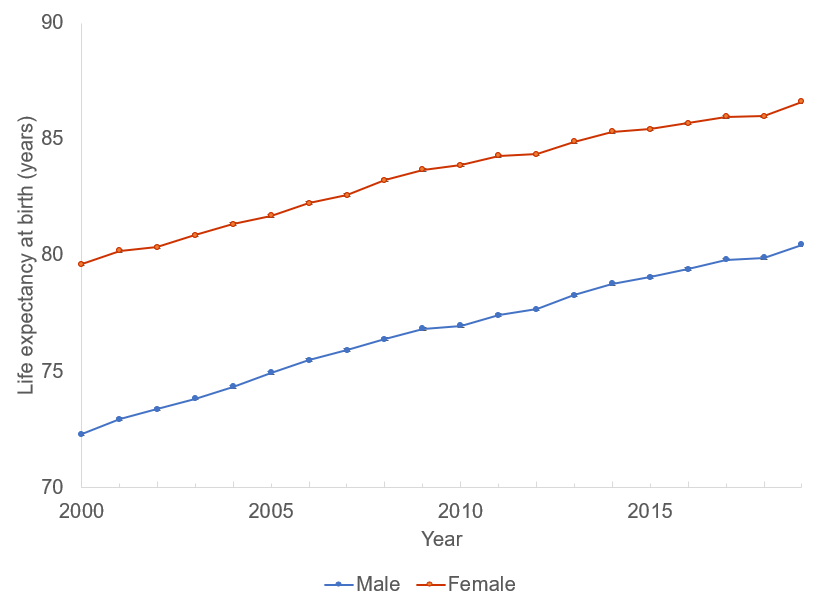


**Supplementary Fig. 1.** Life expectancy change by sex in Korea, 2000-2019: Findings from Korea Statistical Information Service (KOSIS).


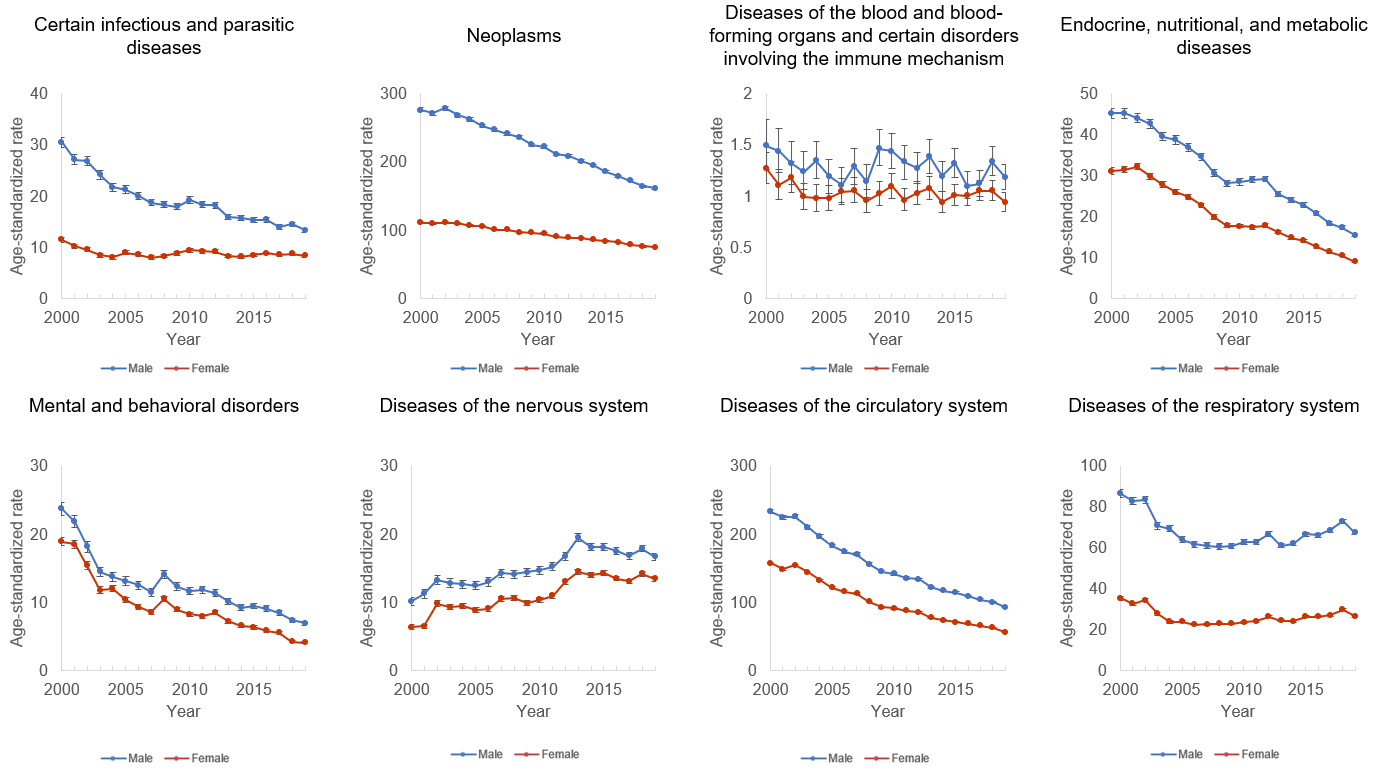


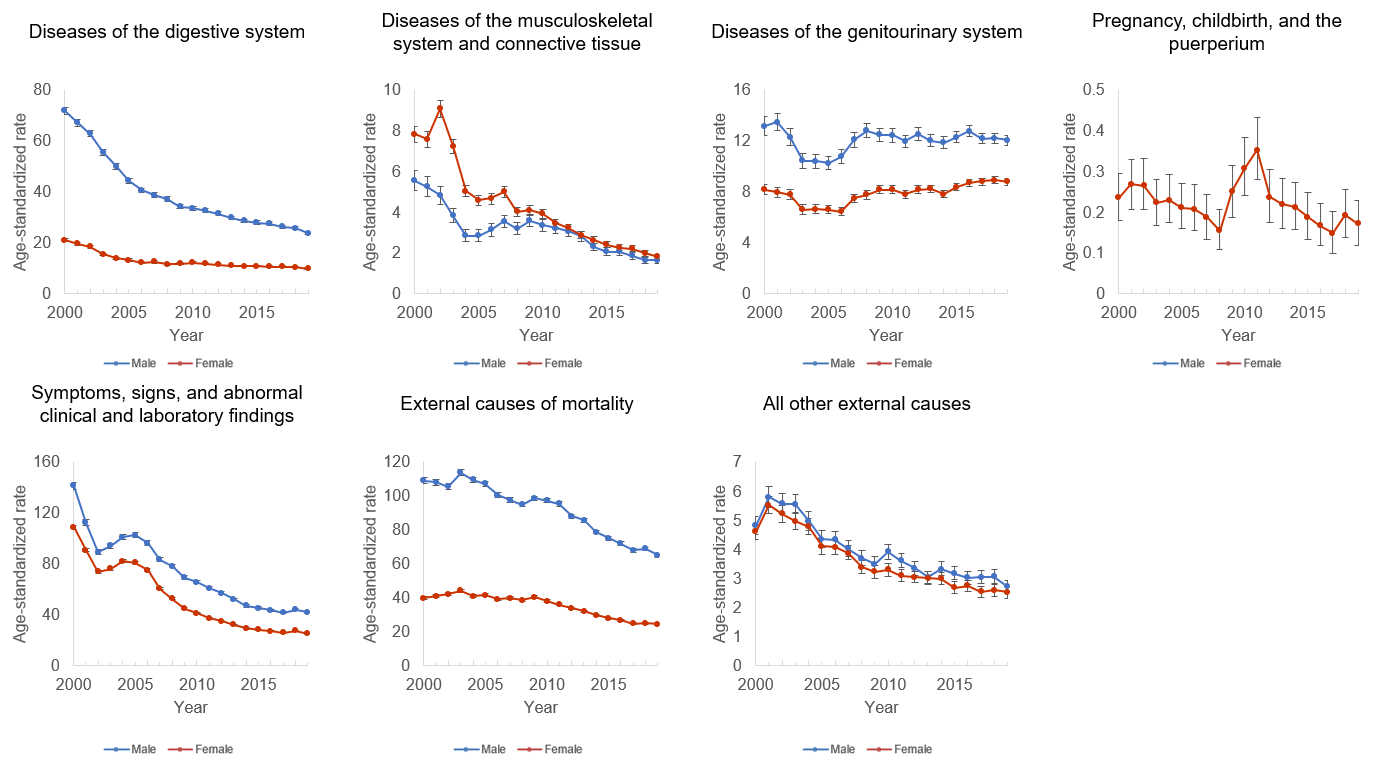


Supplementary Fig. 2. Trends in age-standardized cause-specific mortality by sex during the study period: Findings from the Korean Statistical Information Service (KOSIS).

* The standard population was the total population of Korea in 2010.
